# Widely differing screening and treatment practice for osteoporosis in patients with inflammatory bowel diseases in the Swiss IBD cohort study

**DOI:** 10.1097/MD.0000000000006788

**Published:** 2017-06-02

**Authors:** Solvey Schüle, Jean-Benoît Rossel, Diana Frey, Luc Biedermann, Michael Scharl, Jonas Zeitz, Natália Freitas-Queiroz, Thomas Kuntzen, Thomas Greuter, Stephan R. Vavricka, Gerhard Rogler, Benjamin Misselwitz

**Affiliations:** a Division of Gastroenterology and Hepatology, University Hospital Zurich and University of Zurich, Zurich; b Institute of Social and Preventive Medicine, Lausanne University Hospital, Lausanne; c Division of Rheumatology, University Hospital Zurich and University of Zurich; d Department of Medicine, Division of Gastroenterology, Triemli Hospital, Zurich, Switzerland.

**Keywords:** bone mineral density, inflammatory bowel diseases, osteoporosis, prevention, screening

## Abstract

Low bone mineral density (BMD) and osteoporosis remain frequent problems in patients with inflammatory bowel diseases (IBDs). Several guidelines with nonidentical recommendations exist and there is no general agreement regarding the optimal approach for osteoporosis screening in IBD patients. Clinical practice of osteoporosis screening and treatment remains insufficiently investigated.

In the year 2014, a chart review of 877 patients included in the Swiss IBD Cohort study was performed to assess details of osteoporosis diagnostics and treatment. BMD measurements, osteoporosis treatment, and IBD medication were recorded.

Our chart review revealed 253 dual-energy x-ray absorptiometry (DXA) scans in 877 IBD patients; osteoporosis was prevalent in 20% of tested patients. We identified widely differing osteoporosis screening rates among centers (11%–62%). A multivariate logistic regression analysis identified predictive factors for screening including steroid usage, long disease duration, and perianal disease; even after correction for all risk factors, the study center remained a strong independent predictor (odds ratio 2.3–21 compared to the center with the lowest screening rate). Treatment rates for patients with osteoporosis were suboptimal (55% for calcium, 65% for vitamin D) at the time of chart review. Similarly, a significant fraction of patients with current steroid medication were not treated with vitamin D or calcium (treatment rates 53% for calcium, 58% for vitamin D). For only 29% of patients with osteoporosis bisphosphonate treatment was started. Treatment rates also differed among centers, generally following screening rates. In patients with longitudinal DXA scans, calcium and vitamin D usage was significantly associated with improvement of BMD over time.

Our analysis identified inconsistent usage of osteoporosis screening and underuse of osteoporosis treatment in IBD patients. Increasing awareness of osteoporosis as a significant clinical problem in IBD patients might improve patient care.

## Abstract 2

Supplemental Digital Content is available in the text

## 1 Introduction

Osteoporosis is a clinically relevant and frequent complication in patients with inflammatory bowel disease (IBD).^[1–3]^ Compared to controls, the fracture risk for IBD patients is increased by approximately 40% to 60%.^[4,5]^ Risk factors for osteoporosis and osteopenia in IBD patients include activity and severity of gut inflammation, perianal disease including fistulae, systemic steroid usage, intestinal malabsorption leading to calcium and vitamin D deficiency, low body mass index, and advanced age.^[1,6–22]^

Bone mineral density (BMD) remains a widely accepted parameter to quantify osteopenia and osteoporosis. Dual-energy x-ray absorptiometry (DXA) is normally used to assess BMD. BMD can predict fracture risk^[23,24]^ and a BMD of one standard deviation below the age adjusted mean increases the relative fracture risk by 1.6 to 2.6.^[23]^

Current guidelines recommend screening for osteoporosis in high-risk individuals^[22,25–33]^ (Table 1). For IBD patients recommendations differ in published guidelines by the European Crohn and Colitis Organization, the American College of Gastroenterology (ACG), the American Gastroenterological Association (AGA), and the British Society of Gastroenterology.^[22,25–33]^ Although all guidelines recommend a DXA scan in individuals with significant steroid use and/or recurrent or persistently active disease, each guideline mentions specific additional risk situations. Applicability of these guidelines and compliance with osteoporosis screening for IBD has been insufficiently studied.

**Table 1:**
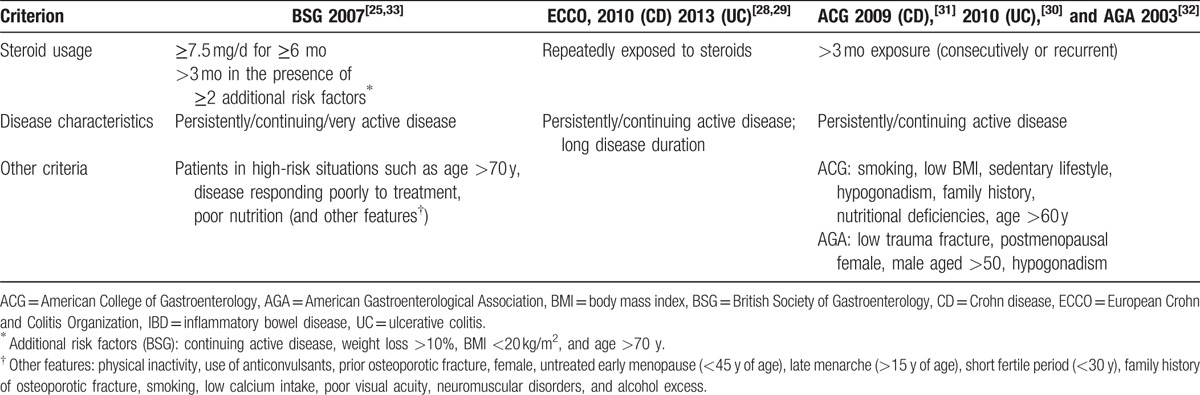
Recommendations regarding osteoporosis screening in IBD patients according to current guidelines.

Adequate treatment can reverse osteoporosis and prevent osteoporotic fractures even in high-risk individuals including postmenopausal women, older men with osteoporosis, or glucocorticoid-treated patients.^[34–36]^ There is general agreement that high-risk individuals with decreased BMD should have adequate dietary calcium intake (1000–1200 mg per day), otherwise calcium supplements should be prescribed. Similarly, an intake of 800 to 1000 international units (IU) of vitamin D per day is recommended.^[35,37,38]^ Furthermore, individuals with osteoporosis should receive osteoporosis medication in addition to calcium and vitamin D treatment (eg, bisphosphonates, parathyroid hormone [PTH] analogues, and estrogens).^[35,37,38]^ During systemic glucocorticoid therapy calcium and vitamin D intake should be adequate, and depending on age, hormonal state, and BMD, additionally bisphosphonates or PTH analogues are recommended.^[35,37–41]^ Several guidelines with similar recommendations exist for the treatment of patients with osteoporosis and IBD.^[1,18,27,42]^ It remains unclear, how these guidelines are applied in clinical practice.

To study screening and treatment of low BMD in IBD patients we used data of the Swiss IBD cohort study (SIBDCS), a prospective long-term study of well characterized IBD patients. Our data indicate divergent screening and treatment rates for IBD patients and possibilities to improve patient care.

## 2 Patients and methods

The SIBDCS is a prospective cohort study of IBD patients. General information regarding the presence of osteopenia/osteoporosis is recorded in the data base but the dates of various DXA scans as well as T scores and Z scores are not. Therefore, a manual review of patient charts was performed in the year 2014. Our chart review covered 4 tertiary care hospitals (providing care from specialists in a large hospital) and 2 secondary care centers. For each available DXA scan T scores and Z scores for hip and lumbar spine were retrieved. Any additional information regarding osteoporosis and osteopenia in the patient chart was also recorded and evaluated as specified below. In addition, information regarding steroid usage ≥10 mg/day, treatment with biologicals (Infliximab, Adalimumab, and Certolizumab pegol) and osteoporosis treatment (calcium, vitamin D, and bisphosphonate medication) was noted. For all treatment parameters, both current usage and any usage within patient history were recorded. For the analysis of the association of steroid treatment with osteoporosis, osteopenia, and normal BMD, treatment information was retrieved from SIBDC data base.

DXA measurements were performed in the femur (femoral neck and/or total hip) and/or lumbar spine. For the T score data were compared to the BMD of a sex-matched young adult reference population while for the Z scores data were compared to an age-, sex-, and ethnicity-matched reference population.^[35]^ In postmenopausal women and in men ≥50 years, osteoporosis and osteopenia were defined by a T score ≤−2.5 and <−1, respectively, in lumbar spine, total hip, or femoral neck.^[35]^ For premenopausal women and younger men, the diagnosis of osteoporosis is not possible on BMD values alone but a Z score of ≤−2 is a helpful parameter.^[35,43]^

A total of 877 patient charts from 6 centers were reviewed for evidence of one or more past DXA scans.^[22]^ Diagnosis or exclusion of osteoporosis and osteopenia was done as described in our previous study,^[22]^ in brief: osteoporosis was defined as T scores ≤−2.5 and Z scores ≤−2, whereas osteopenia was diagnosed at T scores <−1 and >−2.5 and Z scores <−1 and >−2. If scores for both, hip and spine were available, the lowest scores were considered. For our diagnostic procedure the following hierarchy was used: if available, the T score was used. If no T score was available Z score was used. Without information of DXA scores the diagnosis of osteoporosis/osteopenia in the patient chart was considered. For 6 patients an unambiguous reference to a DXA scan was found but no score and no interpretation was available; these patients were only used for the analysis of screening rates but not for statistics about diagnosis and treatment. For the calculations of screening rates evidence for either osteoporosis/osteopenia within the SIBDCS data base or any documentation regarding a DXA scan within patient charts (see above) were taken into account.

### 2.1 Data analysis

For the multivariate analysis the following variables were considered: IBD subtype (Crohn disease [CD] vs ulcerative colitis/indeterminate colitis), gender, last body mass index, last smoking status, steroid use, presence of intestinal stenosis, perianal disease, prior intestinal surgery, presence of malabsorption syndrome, presence of extraintestinal disease manifestations, age at last follow-up, childhood diagnosis of IBD, disease duration, family history of IBD, alcohol consumption more than once a day, sport at least once a week, last Activity Index, and study center, similar to a previous study.^[22]^ For the calculation of the Activity Index, disease activity was normalized to a parameter ranging from 0 (no activity) to 100 (strongest activity). Thereby, for CD patients the Crohn disease activity index was divided by 5; for ulcerative colitis/indeterminate colitis patients the modified Truelove and Witts severity index was divided by .21.^[22]^

Univariate and multivariate logistic regression analyses were used to determine the association of clinical variables with osteoporosis screening in IBD patients. We first performed univariate regressions with each factor mentioned above. We then fit together all variables such that the corresponding *P*-value in univariate regressions was less than .2. A step-wise approach was finally used to select a model with predictors whose *P*-value were less than .157.^[44]^ For this analysis the Stata software was used (StataCorp. 2015. Stata Statistical Software: Release 14. College Station, TX: StatCorp LP).

For the statistical analysis of screening and treatment rates and trends in BMD according to treatments Fisher exact test and a linear regression analysis, respectively, were performed, using appropriate modules of GraphPad Prism, version 6.0d. A *P* value at or below .05 was prospectively defined as significant.

### 2.2 Ethical considerations

The SIBDCS protocol has been approved as a multicenter study by the ethics committee of Zurich County (KEK-ZH). Patients provided written informed consent to data acquisition and analysis during inclusion into the SIBDCS. Data analysis was performed according to the declaration of Helsinki.

## 3 Results

For our analysis, we used a subgroup of SIBDCS, a large prospective cohort study of well-characterized Swiss IBD patients. A chart review was performed in 6 centers. Altogether, data for 877 IBD patients could be retrieved. These patients represent a mixed IBD cohort from tertiary and secondary referral centers with expected epidemiological characteristics regarding age and gender distribution as well as IBD characteristics (Table 2).

**Table 2:**
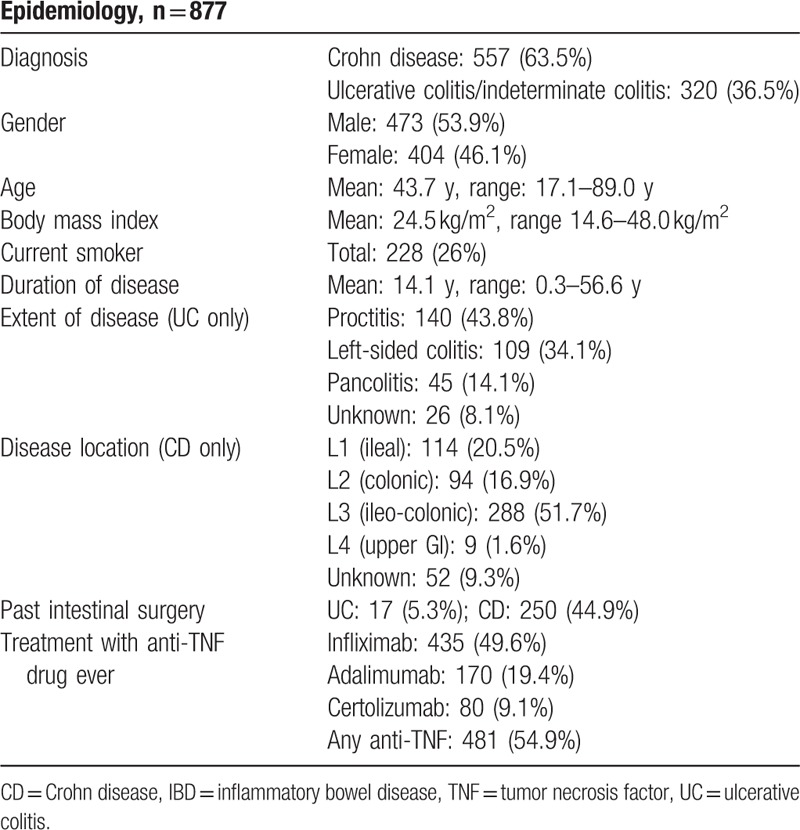
Epidemiological characteristics of our IBD patients in 2014 from 6 Swiss secondary or tertiary health care centers.

### 3.1 Prevalence of osteoporosis screening

**Figure 1:**
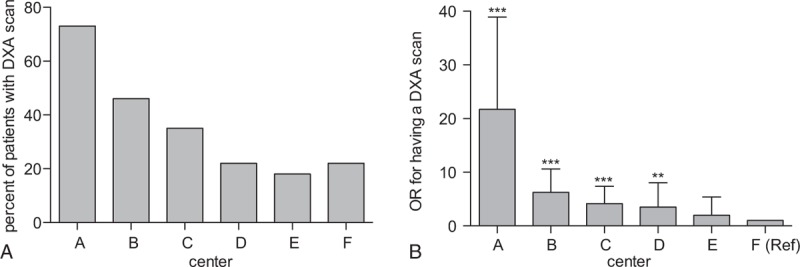
Screening for osteoporosis in 6 Swiss IBD Cohort Study centers from inclusion into the study until year 2014. (A) Screening rates per center. In a conservative approach, screening rates were defined as evidence of osteoporosis/osteopenia in the cohort documentation and/or the patient chart. (B) OR for having a DXA scan in various centers (compare Table 3). Multivariate analysis: ^∗∗∗^*P* < .001, ^∗∗^*P* < .01, ^∗^*P* < .05. DXA = dual-energy x-ray absorptiometry, IBD = inflammatory bowel disease, OR = odds ratio.

In 259 of the 877 patients (30%), osteoporosis screening was performed. Screening rates differed strongly between centers ranging from 11% to 62% (Fig. 1). For example, in center A, 90 out of 146 patients (62%) have had a DXA scan; in center B, 83 out of 231 (36%) were screened by DXA whereas in center F only 25 out of 237 patients (11%) have had a DXA scan. Overall, screening rates for osteoporosis tended to be slightly higher in tertiary referral centers compared to secondary centers (30.2% compared to 24.5%, not significant). However, pronounced differences were also observed within the group of tertiary care centers (Fig. 1, centers A, B, E, F).

In a multivariate analysis considering multiple risk factors^[22]^ (see Patients and methods), the study center remained a strong independent and significant predictive factor for the performance of a DXA scan (Table 3). The following clinical variables were significantly associated with performance of a DXA scan: presence of perianal disease (odds ratio 1.52; confidence interval [CI]: 1.04–2.2; *P* = .032) and usage of any steroid at last visit, including budesonide (odds ratio 2.2; CI: 1.5–3.2; *P* < .001). Treatment with budesonide on its own (instead of all steroids) was also significantly associated with osteoporosis screening; however, the association of all steroids including budesonide with osteoporosis screening was stronger (ie, resulted in better model characteristics; not shown). Age at diagnosis, disease duration, gender, and presence of primary sclerosing cholangitis did not significantly influence the decision to screen in this multivariate analysis.

**Table 3:**
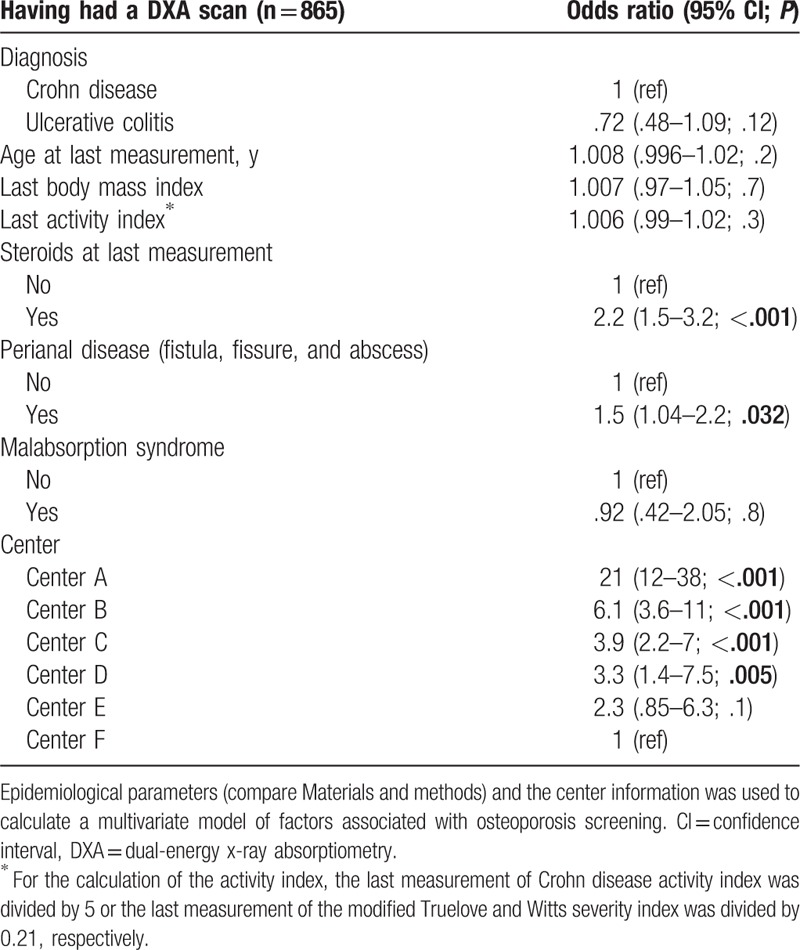
Multivariate model for having a DXA scan.

These results suggest that clinicians considered clinical parameters for their decision to order a DXA scan but clinical practice differed strongly among centers.

### 3.2 Prevalence of osteoporosis and osteoporosis risk factors

**Figure 2:**
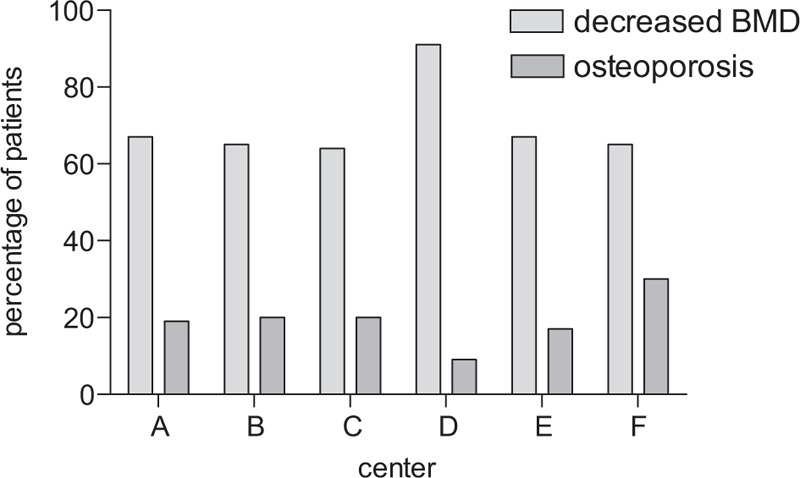
Fraction of dual-energy x-ray absorptiometry (DXA) scans diagnostic for osteopenia or osteoporosis in 6 Swiss study centers from inclusion into the study until year 2014. Percentage of DXA scans diagnostic for osteoporosis or osteopenia are shown. Rates of positive findings did not differ significantly (Chi-square test).

Overall, 169 of 877 patients (19.3%) had documented decreased BMD. When only the 253 patients with available DXA scans were considered, osteopenia was found in 57% and osteoporosis in 20%. Looking at the different centers separately, among patients with DXA scans rates for reduced BMD ranged from 43% to 82% and for osteoporosis from 9% to 30% (Fig. 2). Patients aged ≥50 years (103 out of 253) showed higher rates of osteoporosis compared to younger patients (29.1% vs 12%, *P* = .001); however, the rates of osteopenia did not differ (45.6% in patients ≥50 years vs 45.3% in patients <50 years). In patients with a disease duration of ≥15 years (141 out of 253), rates of osteopenia and osteoporosis did not differ significantly (osteopenia: 49.6% vs 40.2%; osteoporosis: 20.6% vs 16.1%, ns).

Table 4 provides a comparison of patients with osteoporosis, osteopenia, and normal BMD. Patients with osteoporosis were elder, more likely to suffer from CD and more likely to be male compared to patients with osteopenia and normal BMD, while disease duration and prevalence of primary sclerosing cholangitis did not differ significantly (Table 4). Rates of steroid treatment at last visit were highest for osteoporosis (79.2%), intermediate for osteopenia (62.6%), and lowest for normal BMD (42.2%; *P* < .001). Rates of budesonide treatment at last visit also differed according to BMD but this trend failed to reach significance. Interestingly, the percentage of positive DXA scans was not related to the screening rate (compare Figs. 1 and 2; no significant association in a Spearmen correlation analysis).

**Table 4:**
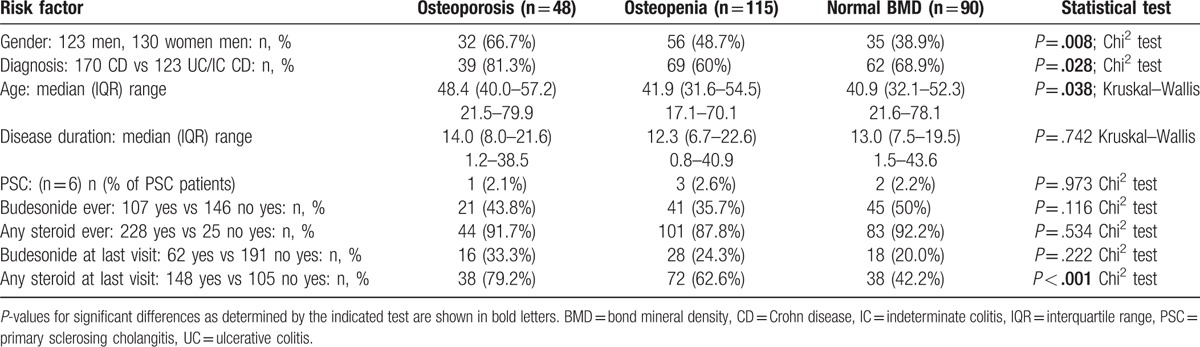
Risk factors for osteoporosis in 253 patients with known BMD.

### 3.3 Treatment of osteoporosis

**Figure 3:**
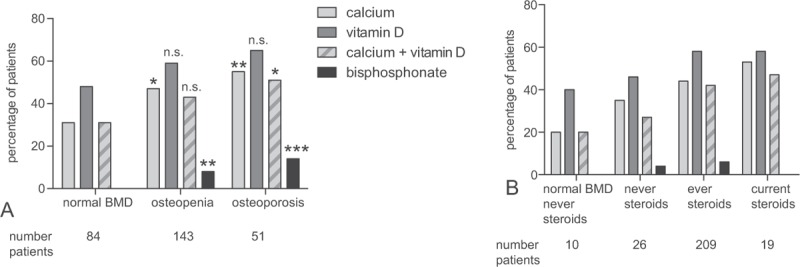
Osteoporosis treatment. (A) Percentage of patients with osteoporosis treatment at the time of chart review according to results of DXA scans. For the statistical analysis patients with osteoporosis/osteopenia were compared to patients with normal BMD. Fisher exact test: ns, ^∗^*P* < .05, ^∗∗^*P* < .01, ^∗∗∗^*P* < .001. (B) Treatment in patients depending on their history of steroid therapy. For the statistical analysis patients which never received steroids were compared to patients with current or any steroid treatment. No significant differences were found. BMD = bond mineral density, DXA = dual-energy x-ray absorptiometry, ns = not significant.

Treatment of reduced BMD differed within our cohort. This analysis was restricted to 253 patients with known BMD. Overall, 106 of 253 patients (42%) were treated with calcium supplementation, 140 (55%) with vitamin D, and 14 (6%) with bisphosphonates at the time of chart review. Treatment differed according to BMD: 28/51 (55%) of patients with osteoporosis, 67/143 (47%) of patients with osteopenia, and 26/84 (31%) of patients with normal BMD were treated with calcium (*P* = .013, Chi square test for the whole group, compare Fig. 3A). Similar but nonsignificant effects on treatment rates for vitamin D were recorded and 33/51 (65%) of patients with osteoporosis, 84/143 (59%) of patients with osteopenia, and 40/84 (48%) of patients with normal BMD received vitamin D supplementation (*P* = .112). The fraction of patients that did not receive any osteoporosis treatment at the time of chart review was 27% for osteoporosis and 36% for osteopenia.

When the complete treatment history of the patient was considered, rates for patients ever treated with calcium/vitamin D increased to 92%/90% for patients with osteoporosis, 83%/87% for patients with osteopenia, and 61%/70% for patients with normal BMD.

Centers with higher rates of osteoporosis screening also showed higher rates for osteoporosis treatment. For the 3 centers with the highest screening rates (center A–F, Fig. 1) treatment rates followed screening rates with calcium/vitamin D medication in 54%/71% in center A, 39%/54% in center B, and 27%/34% in center C. The trend remained robust if subgroups of patients with osteoporosis or osteopenia were considered.

Patients currently treated with steroids tended to receive calcium and vitamin D supplementation slightly more frequently than patients without steroids (10/19; 53% for calcium; 11/19; 58% for vitamin D, not significant; Fig. 3B). However, a considerable fraction of patients with normal BMD and no previous steroids nevertheless received replacement therapy (2/10 for calcium, 4/10 for vitamin D).

Bisphosphonate treatment was most frequently applied to patients with osteoporosis: 7 out of 51 osteoporosis patients (14%) received bisphosphonates at the time of the chart review. In addition, 11 out of 143 patients (8%) with osteopenia but no patient with normal BMD received bisphosphonate treatment at this time. For 13 patients bisphosphonate treatment had been started but discontinued and 15 out of 51 (29%) of patients with osteoporosis, 19 out of 143 with osteopenia (13%), and 1 out of 84 patients with normal BMD (1%) had ever received bisphosphonates. The reasons why treatment was discontinued were not evaluated.

### 3.4 Multiple DXA scans and improvement of BMD

**Figure 4:**
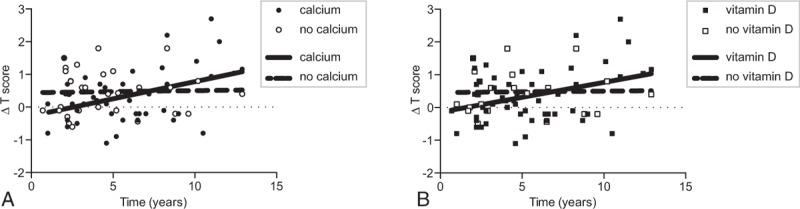
Improvement of dual-energy x-ray absorptiometry (DXA) results of the lumbar spine upon treatment with vitamin D or calcium. (A) Changes in T scores of the spine over time with and without calcium treatment (*R*^2^ = .17, *P* = .004, linear regression analysis). For comparison patients without calcium treatment are shown. (B) Changes in T scores with and without vitamin D treatment (*R*^2^ = .13, *P* = .007).

Among the 259 IBD patients with DXA screening 129 (50%) received 1 DXA scan; 72 (28%) were tested twice, 26 (10%) 3 times, and 18 (7%) and 14 (5%) 4 or more times, respectively (Figure S1). Overall, for all patients with multiple DXA scans we note a slight improvement over time in T scores for the hip and the spine in a linear regression analysis (spine: slope: .06/year, CI: .0099–.12, *P* = .02; hip: slope: .04/year, CI: −.0002–.081, *P* = .051, not shown). However, in the subgroup of patients with calcium or vitamin D supplementation T scores for spine improved significantly (calcium: slope: .1/year, CI: .036–.17, *P* = .004; vitamin D: slope: .091/year, CI: .026–.16, *P* = .007, vitamin D and calcium: slope .089/year, CI: .028–.15, *P* = .005; Fig. 4A and B). In contrast, for patients without calcium and vitamin D supplementation spinal T scores did not increase significantly over time. Similar results were obtained for T scores of the hip (not shown). No significant improvement in serial DXA scans of 8 patients with bisphosphonate treatment was noted, but the low number of patients in this subgroup limits our conclusions.

Treatment with any tumor necrosis factor inhibitor (Infliximab, Adalimumab, or Certolizumab) was not associated with improvements in T scores for hip or spine in contrast to 2 previous studies.^[45,46]^

## 4 Discussion

In our study, we address clinical practice of osteoporosis screening and treatment. We found widely differing screening rates among different IBD referral centers. Furthermore, osteoporosis treatment frequently did not follow recommendations, most profoundly in centers with low screening rates. Our data thus indicate low awareness of osteoporosis as an important medical problem for IBD patients. However, the subset of patients with calcium or vitamin D medication significantly improved their BMD over the course of treatment, in turn indicating, that appropriate evaluation for and treatment of osteopenia/osteoporosis may ultimately translate into clinical benefit for patients with IBD.

In 253 patients tested with DXA scans, rates of osteopenia and osteoporosis were 57% and 20%, respectively. These numbers are well in agreement with previous studies, reporting osteopenia and osteoporosis rates of 34% to 78% and 13% to 42%, respectively.^[20,22,47–52]^ Although the rate of low BMD of a given cohort will depend on patient and disease characteristics, our data confirm osteoporosis and osteopenia as prevalent problems in IBD patients. Our study also confirms the important role of steroid treatment for osteoporosis in IBD patients (Table 4).

Screening rates for osteoporosis varied remarkably among the 6 study centers, ranging from 11% to 62%. All patients were treated by gastroenterologists specialized in IBD care, either in large academic centers or in large private practices. Nevertheless, the study center remained a strong and significant predictor for osteoporosis screening in univariate and multivariate analyses. Clinical variables such as steroid usage, disease duration, and presence of perianal disease were further predicting factors. Interestingly, even though the rates of DXA scans strongly differed among centers, the fraction of positive DXA scans (ie, with a diagnosis of osteoporosis or osteopenia) did not differ significantly (Fig. 2). Our analysis cannot formally distinguish between over usage and under usage of DXA scans. However, our data suggest that either the policy of a given center, awareness of osteoporosis, and/or availability of DXA scans strongly influenced clinical management of IBD patients regarding bone health.

Current guidelines agree that DXA scan should be recommended in individuals with significant steroid use longer than 3 or 6 months or recurrent and/or persistently active disease. However, compliance to most guidelines cannot be formally tested since no formal threshold for disease duration or “persistent” or “continuous” disease activity is defined by European Crohn and Colitis Organization or British Society of Gastroenterology guidelines.^[25,26,28,29,33]^ Guidelines of AGA and ACG provide objective criteria^[32]^ but none of these are specific to IBD patients. In 1 study, screening criteria mentioned by AGA and ACG guidelines did not predict low BMD in subsequent DXA scans.^[53]^

Steroid usage is mentioned by all guidelines but details of recommendations differ. However, during our chart review extracting steroid dosage over time proved to be time consuming (up to 1 hour per patient) and frequently an area under the curve could not be reconstructed with confidence. Considering complex patient histories or treatment in different clinical settings, we suspect that these limitations are not specific to our study and any screening recommendation based on duration or frequency of past steroid usage will be hard to implement in a rigorous manner.

Awareness of osteoporosis was directly tested in 1 previous study demonstrating low familiarity of physicians of AGA with guidelines regarding osteoporosis screening and treatment in IBD patients.^[54]^ According to another prospective study, increasing physician's awareness of osteoporosis guidelines can in turn improve screening rates in IBD patients^[55]^: guidelines of ACG were sent to members, prompting additional DXA scans as well as increased familiarity in osteoporosis treatment, potentially preventing fractures in IBD patients.

Taken together, heterogeneity in clinical practice (as indicated by our study) might also reflect ambiguity and diversity in current guidelines. Overutilization and under usage of DXA scans will clearly limit cost-efficiency of osteoporosis screening and guidelines easily applicable in clinical practice would be desirable.

Treatment rates regarding calcium and vitamin D generally followed screening rates. A diagnosis of reduced BMD increased the likelihood of treatment with vitamin D and/or calcium. However, 27% and 36% of patients with osteoporosis and osteopenia, respectively, did not receive treatment. Our data thus reveal partial noncompliance with osteoporosis treatment guidelines in Switzerland. Although the vast majority of patients (almost 92%) with a DXA scan diagnostic for osteoporosis had received vitamin D and calcium in the past, only 55% of patients received calcium and 65% received vitamin D at the time of our chart review. Low treatment rates were also described in previous studies^[53,56]^ (treatment rates for calcium and/or vitamin D of 59%–63.5% in IBD patients with low BMD).

Treatment with calcium and vitamin D is likely beneficial for IBD patients with low BMD and an increase in T scores in sequential DXA scans was noted in patients with calcium and vitamin D treatment. Similar effects were described in a previous randomized, placebo controlled study with 60 IBD patients.^[57]^

In our study, only 29% of all patients with osteoporosis were ever treated with bisphosphonates and for only 14% of patients this drug was part of the current treatment regiment, pointing to a relevant underutilization of this very efficient osteoporosis medication.^[34–36]^ Treatment with bisphosphonates can reduce the incidence of spine and hip fractures by 33% to 50% over 3 years in osteoporotic postmenopausal women.^[32,35,36]^ Underuse of bisphosphonates is unlikely due to financial constraints since these costs will be reimbursed by the universal Swiss public health insurance system. Besides bisphosphonates other powerful osteoporosis treatments are available including calcitonin, estrogens, PTH analogues, and denosumab.^[35]^ However, prescription of these modern therapies was noted in only 3 out of 169 patients with low BMD.

Strengths of our study include the high level of patient data available. Furthermore, to the best of our knowledge, our study is the first comparing clinical practice regarding screening and treatment rates among individual centers. Our study has several limitations:

(i) The retrospective study design.
(ii) Our study population was recruited in secondary and tertiary referral centers and might not be representative for the Swiss population affected by IBD.
(iii) Our analysis was limited to results of DXA scans and fractures were not considered.
(iv) We did not analyze biochemical markers for systemic inflammation^[21,58]^ or intestinal inflammation such as calprotectin, bone turnover,^[47,50,58]^ or genetic markers for osteoporosis^[59]^ as done in some previous studies.
(v) We did not systematically assess comorbidities of our patients besides IBD; however, only 10.7% of all patients and 11% of patients with DXA scans were older than 65 years and the influence of comorbidities might be limited.

## 5 Conclusion

Our analysis identified inconsistent usage of osteoporosis screening and underuse of osteoporosis treatment with calcium, vitamin D, and bisphosphonates in IBD patients. Screening and treatment rates strongly differed among centers and opportunities for improving treatment remain in many centers. Treatment with calcium and vitamin D improved BMD in DXA scans. Better physician awareness regarding osteoporosis might thus improve bone health of IBD patients.

## Acknowledgments

The authors thank Swiss National Science Foundation to BM (Grant No. 32473B_156525) and the Swiss IBD Cohort (Grant No. 3347CO-108792) for the support.

## Supplementary Material

Supplemental Digital Content
