# Supplementary material for: Widely differing screening and treatment practice for osteoporosis in patients with inflammatory bowel diseases in the Swiss IBD cohort study
Source: Medicine (Baltimore). 2017 Jun 2;96(22):e6788. doi: 10.1097/MD.0000000000006788 (PMC5459696; doi:10.1097/MD.0000000000006788)

Figure S1. Number of DXA scans performed in patients of the Swiss IBD Cohort Study screened for osteoporosis. Of 259 patients with evidence of a DXA scan the fraction of patients with 1 or more scans from inclusion into the study until year 2014 is indicated by the color.


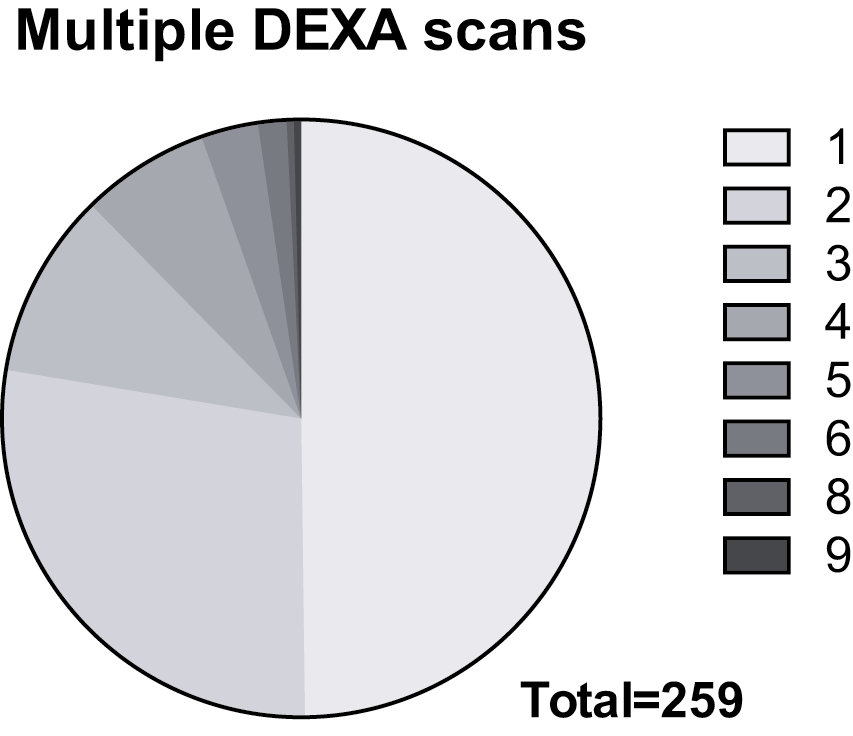

Supplement: Supplemental Digital Content [file medi-96-e6788-s001.doc]
